# Interactive effects of UV radiation and water deficit on production characteristics in upland grassland and their estimation by proximity sensing

**DOI:** 10.1002/ece3.9330

**Published:** 2022-09-23

**Authors:** Petr Holub, Karel Klem, Barbora Veselá, Kateřina Surá, Otmar Urban

**Affiliations:** ^1^ Global Change Research Institute of the Czech Academy of Sciences Brno Czech Republic; ^2^ Mendel University in Brno Brno Czech Republic

**Keywords:** drought, grassland, infrared thermography, nitrogen, precipitation, spectral reflectance, UV radiation

## Abstract

An increase in extreme weather and changes in other conditions associated with ongoing climate change are exposing ecosystems to a very wide range of environmental drivers that interact in ways which are not sufficiently understood. Such uncertainties in how ecosystems respond to multifactorial change make it difficult to predict the impacts of environmental change on ecosystems and their functions. Since water deficit (WD) and ultraviolet radiation (UV) trigger similar protective mechanisms in plants, we tested the hypothesis that UV modulates grassland acclimation to WD, mainly through changes in the root/shoot (R/S) ratio, and thus enhances the ability of grassland to acquire water from the soil and hence maintain its productivity. We also tested the potential of spectral reflectance and thermal imaging for monitoring the impacts of WD and UV on grassland production parameters. The experimental plots were manipulated by lamellar shelters allowing precipitation to pass through or to be excluded. The lamellas were either transmitting or blocking the UV. The results show that WD resulted in a significant decrease in aboveground biomass (AB). In contrast, belowground biomass (BB), R/S ratio, and total biomass (TB) increased significantly in response to WD, especially in UV exclusion treatment. UV exposure had a significant effect on AB and BB, but only in the last year of the experiment. The differences in the effect of WD between years show that the effect of precipitation removal is largely influenced by the potential evapotranspiration (PET) in a given year and hence mainly by air temperatures, while the resulting effect on production parameters is best correlated with the water balance given by the difference between precipitation and PET. Canopy temperature and selected spectral reflectance indices showed a significant response to WD and also significant relationships with morphological (AB, R/S) and biochemical (C/N ratio) parameters. In particular, the vegetation indices NDVI and RDVI provided the best correlations of biomass changes caused by WD and thus the highest potential to remotely sense drought effects on terrestrial vegetation.

## Graphical abstract

The manuscript focuses on the interactive impacts of water deficit and UV radiation on the production of mountain grassland. We tested the hypothesis that UV modulates grassland acclimation to drought stress, mainly through changes in the root/shoot ratio, enhances the ability of grassland to acquire water from the soil, and hence affects its productivity. Applied spectral reflectance and thermal imaging techniques represent innovative methodological approaches having a potential for further upscaling and application at large scales.

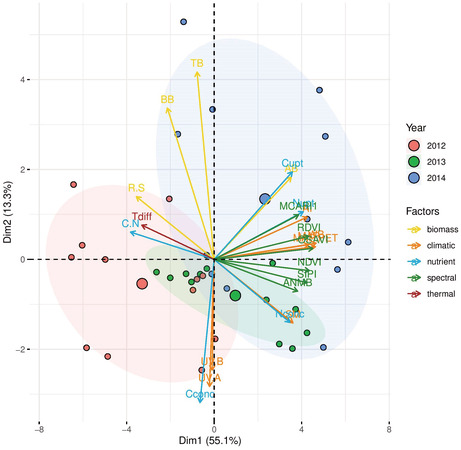

## 1 INTRODUCTION

It is widely accepted that the frequency and magnitude of extreme weather events will increase with global climate change (Dai, 2011; IPCC, 2021). The effects of extreme weather events on vegetation and ecosystem functioning are likely to be much greater than the effects of long‐term trends in mean temperature and precipitation (Easterling et al., 2000; Hoover et al., 2014; Jentsch & Beierkuhnlein, 2008). Such changes will affect numerous soil, plant, and ecosystem properties, including the productivity and biodiversity of grasslands (Fay et al., 2008; Knapp et al., 2002; Kreyling et al., 2008). Increasing extremity, as well as the frequency of such weather events, is inevitably associated with interactive effects of several climatic drivers on the physiology and productivity of vegetation. These effects have not yet been explored. Interactive effects can range from antagonistic to synergistic depending on the intensity and duration of stress conditions. A typical example of an interactive effect which we explore in the present work is the combination of UV radiation and drought (Jansen et al., 2022). Low UV doses usually induce defense mechanisms at morphological (Robson et al., 2015) and/or biochemical levels (Rodríguez‐Calzada et al., 2019) and contribute thus to the mitigation of negative drought effects. However, additive or multiplicative effects are often observed at high UV doses and under severe drought conditions (Bandurska et al., 2013; Jansen et al., 2022). The direction of interactive effects usually depends on the intensity of the dominant driver, and there is often a marked nonlinearity in plant responses, sometimes with unexpected “tipping points” (Jansen et al., 2022; Wang et al., 2022). Complex responses, including hermetic effects, may be involved even at low doses of stress factors (Erofeeva, 2022). However, current experiments do not sufficiently allow evaluation of the entire suite of interactions between climatic drivers, particularly at the full range of ecologically relevant doses and at the whole ecosystem level. This would require a bewildering range of variables including species composition and soil as well as climatological variables. To advance our understanding of such complex interactions, a combination of two methodological approaches seems necessary: (i) to synthesize data from multiple experiments covering wide ranges of natural conditions and interannual variabilities (Jansen et al., 2022) and (ii) to develop remote sensing methods allowing the evaluation of plant responses to a complex set of conditions of their natural environments (e.g., Yuan et al., 2019).

The relationships between vegetation productivity and single climatic components, mainly precipitation and temperature, have been studied by several authors. In most studies on grasslands, the aboveground net primary productivity is positively correlated with mean annual precipitation (e.g., Fay et al., 2008; Qin et al., 2018; Yahdjian & Sala, 2006). The responses to precipitation change should be, however, considered along with the temperature. The ecosystems adapted to higher temperatures have developed the strategy of conservative water use in response to higher evaporative demands, which is not the case for ecosystems adapted to colder conditions. For example, in a synthesis of 83 studies, Wilcox et al. (2017) have shown that cooler mountain ecosystems are more sensitive to changes in precipitation. For the same reasons, the effect of precipitation decline should be considered more detrimental for ecosystems adapted to colder conditions when combined with temperature increases. A key adaptation to drought may be seen in the relationship between belowground and aboveground production (e.g., Bakker et al., 2006; Ibrahim et al., 1997; Qaderi et al., 2006). Such adaptation results in increasing values of root‐to‐shoot ratio (R/S) and is a well‐known drought avoidance strategy (e.g., Li et al., 2021; Rodrigues et al., 1995). However, published data also show that water availability may be a limiting factor for BP and alters the fraction of BP in total production (e.g., Frank, 2007; Xu et al., 2012). Similar adaptive responses of BP or R/S can be induced also by enhanced UV radiation, particularly UV‐B (Robson et al., 2015; Uchytilová et al., 2019). Another common pathway in which the drought and UV radiation induce or use similar protective mechanisms is the antioxidant defense system (Cechin et al., 2008). This mechanism includes either the nonenzymatic as flavonoids and carotenoids (Klem et al., 2015) or the enzymatic constituents (Basu et al., 2010) contributing to the detoxification of reactive oxygen species. Another possible “crosstalk” between the response to drought and UV radiation is represented by stomata regulation (Yang et al., 2020). Although the response of stomatal conductance to UV radiation is contrary to drought quite variable, ranging from negative to positive effects, the interactive effects of UV radiation and drought are mostly negative with the slightly enhanced effect of drought, indicating rather additive (or synergistic) than antagonistic interaction (Jansen et al., 2022).

Since all these adaptive mechanisms play a role in the regulation of water stress (Gitz & Liu‐Gitz, 2003) as well as UV‐B impacts (Ibañez et al., 2008), a wide range of interactive effects between UV radiation and drought may be assumed. However, the data concerning the interaction between UV radiation and drought as well as the implications on plant metabolic processes are controversial, indicating diverse interactive effects ranging from antagonistic to synergistic (Jansen et al., 2022). For example, ameliorating effects of drought on UV‐B sensitivity have been reported by Sullivan and Teramura (1990). In addition, it has been suggested that under multiple stress conditions, exposure to UV radiation moderates the effects of drought (Cechin et al., 2008; Schmidt et al., 2000) or that each stress factor seems to bring about some adaptive effect to reduce the damage caused by the other one (Alexieva et al., 2001; Hofmann et al., 2003; Zhang et al., 2020). In contrast, other findings show synergistic (additive) interactions, resulting in enhanced sensitivity to UV radiation under reduced water availability (Lu et al., 2009; Tian & Lei, 2007). A better understanding of such variability requires a focus on the effect of both factors in the context of stress intensity. For these purposes, multiannual field experiments covering the natural interannual variability of drought and UV may provide the necessary analytical power.

Due to the complexity of interactions with other factors, which show high temporal and spatial variability, estimation of drought and UV radiation impacts on the ecosystem productivity is challenging. The primary possibility for repeatedly assessing drought impacts on large areas is represented by proximity or remote sensing approaches. In particular, thermal infrared imaging and spectral reflectance can be applied to detect ecosystem functions and productivity. Thermal infrared imaging primarily enables the detection of changes in leaf temperature caused by stomatal movements (Grant et al., 2006), a signal of early‐stage response of plants to drought and UV impact (Jansen & van den Noort, 2000; Jones et al., 2009). Generally, reduction in stomatal conductance leads to reduced transpiration and a subsequent increase in leaf temperature. Thermal imaging can detect even subtle changes in leaf temperature and thus has an important role to play in revealing interactive effects of drought and UV on stomatal conductance and their spatiotemporal variabilities.

On the contrary, spectral reflectance is based on biophysical parameters of vegetation such as leaf area, biomass allocation, and/or chlorophyll and water contents. These parameters are more stable in time, and their changes represent late‐stage responses of plants to drought or UV exposure. Such responses are associated with a certain degree of damage (decreases in biomass and chlorophyll content), accumulation of oxidative damage products, and/or acclimation responses including increases in flavonoid content or leaf thickness. Hence, the estimation of drought and UV impacts may be based on spectral reflectance indices, which identify changes in biomass (NDVI, Aparicio et al., 2000), conversion of xanthophyll cycle carotenoids (PRI, Elsheery & Cao, 2008), water content (WI, Peñuelas et al., 1997), accumulation of oxidized phenolic compounds (BPI, Peñuelas et al., 2004), and/or accumulation of UV screening compounds (Klem et al., 2012). Spectral reflectance thus allows detection of different types of response compared with the thermal infrared imaging; however, as an indirect type of measurement, the results can be modulated by other factors (e.g., nutrient availability, light conditions, and canopy structure; Hatfield et al., 2008).

The main objectives of the present study were (i) to investigate the effects of interannual natural variability in water deficit (WD), UV radiation (UV), and their combination (WD + UV) on the above‐ and belowground productivity of mountain grassland and (ii) to evaluate the applicability of infrared thermal imaging and spectral reflectance techniques in the detection of plant responses to such conditions. We formulated the following hypotheses: (1) natural doses of UV alleviate the negative effects of WD on plants and ecosystems through an enhancement in the R/S ratio and improved ability to acquire water from the soil and (2) thermal infrared imaging, related to stomatal conductance of plants, is sensitive enough to detect changes induced by both WD and UV conditions, whereas spectral reflectance indices are mainly sensitive to detect changes in biomass accumulation and chlorophyll content—that is, traits primarily affected by WD.

## 2 MATERIALS AND METHODS

### 2.1 Site description

The experimental plots were established in a mountain *Nardus* grassland (class *Calluno‐Ulicetea*, alliance *Violion‐caninae*) situated in the Moravian‐Silesian Beskydy Mts., the Czech Republic (near the Ecological Experimental Study Site Bílý Kříž, altitude 855 m, latitude 49°30′ N, 18°32′ E). The site is characterized by a mean long‐term annual temperature of 6.8°C and precipitation of 1318 mm. The geological bedrock is formed by Spodo‐dystric cambisol on Flysch Godulian sandstone. The experimental area was selected to represent a homogeneous segment of vegetation, allowing proper experimental design with randomized replications in the block. Narrow‐leaved grasses *Nardus stricta, Festuca rubra*, *Agrostis capillaris, Holcus mollis*, *Avenella flexuosa*, and *Carex pilulifera* are the major components of the vegetation in this grassland. The most frequented forbs were *Hypericum maculatum, Rumex acetosa, Veronica officinalis, Potentilla erecta*, and *Hieracium lachenalii*. The grassland is regularly mowed once a year in July.

### 2.2 Experimental design

The field experiment was carried out in a two‐factorial design manipulating WD and UV radiation during 2012–2014. The design consisted of 12 plots, each 2 × 1.5‐m in size, with all factorial combinations replicated three times. To determine the responsiveness of mountain grassland vegetation to drought and UV radiation, six transparent shelters (3 × 2‐m in size) were constructed as a wood frame covered with transparent acrylic lamellas (thickness of 3 mm; Quinn Plastics) with different UV‐A and UV‐B transmittance that had a 20° inclination. The first type (UVT Solar) transmitted more than 90% of incident UV‐A and UV‐B radiation, whereas the second one (Quinn XT) filtered UV‐B radiation and a large part of UV‐A.

The shelters were installed in all plots every year from April, just after snowmelt, until mowing in July. The exclusion of rainfall in dry plots was provided by modifying the position of the acrylic lamellas so that they overlapped in the direction from the top. In this case, the water intercepted by the lamellas was channeled using a gutter out of the experimental area. On the contrary, lamellas in wet treatments overlapped in the direction from the bottom and thus allowed complete penetration of rainfall to the experimental plot. Thus four treatments were maintained: (i) control with ambient precipitation and UV exclusion (C); (ii) water deficit and UV exclusion (WD); (iii) ambient precipitation and UV exposure (UV); and (iv) water deficit and UV exposure (WD + UV). This set of treatments enables the estimation of the effect of UV, WD, and their combination in years with different precipitation and evaporative demands, the interpretation of the complex interactions between drought and UV radiation, and the understanding of the potential mechanisms behind the interactive effects such as changing R/S ratio, or biochemical changes related to altered C/N ratio. Induced drought period (IDP) was manipulated in spring, this being the maximal growth of vegetation (Table 1). A 0.2‐m‐wide trench was dug and sheathed with plastic foil to separate the soil of the roofed areas from the neighboring soil. A 0.25‐m‐wide zone beneath the edge of the shelters was excluded from all measurements and samples. The distance between the shelter and soil surface was 1.3 m to maximize air movement and minimize temperature and relative humidity artifacts. Heating under the shelters was minimized by the gaps between individual lamellas, through which the warm air rose upwards and escaped.

**TABLE 1:** Amount of precipitation (P; mm), aridity index (AI), ratio between precipitation and potential evapotranspiration (P/PET) in control and WD treatment, UV‐A (kJ m^−2^) and UV‐B (kJ m^−2^) in control and UV treatment during vegetation season (from April to measurement day—MD).

Year	2012		2013		2014	
Treatment	Control	WD	Control	WD	Control	WD
IDP	10 May–10 July		15 May–3 July		21 May–3 July	
MD	July 10	July 10	July 3	July 3	July 3	July 3
P	279	67	279	135	431	175
AI	1520	342	1645	796	3340	1357
P/PET	1.04	0.23	1.50	0.73	2.03	0.82
Treatment	Control	UV	Control	UV	Control	UV
UV‐A	93.4	402.7	93.6	320.5	81.9	340.0
UV‐B	4.5	25.9	3.3	15.6	3.8	25.7

Abbreviations: IDP, induced dry period.

### 2.3 Climatic parameters

**FIGURE 1:**
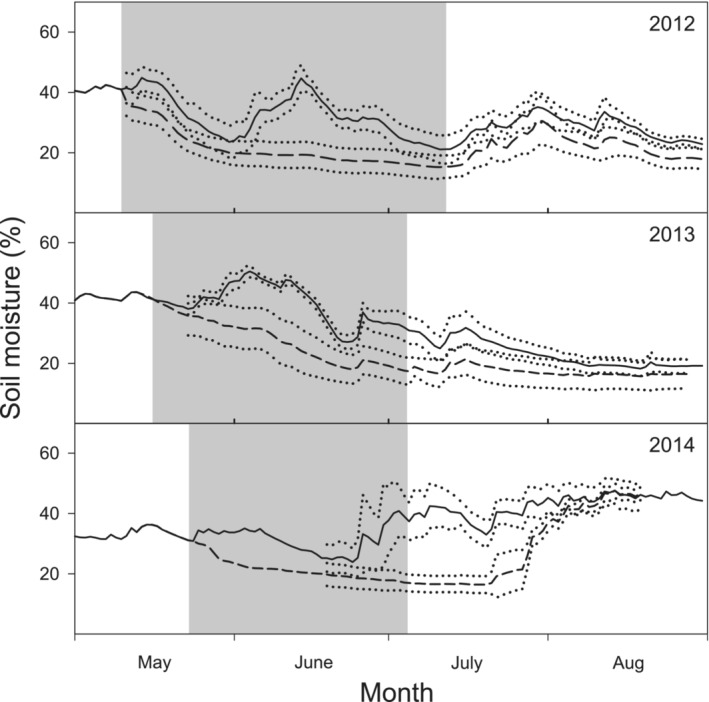
Dynamics of daily mean soil moisture in ambient (C; solid line) and water deficit (WD; dashed line) treatments over three experimental seasons 2012–2014. Gray background indicates the induced drought period.

Precipitation and air temperature were measured every 30 min at a meteorological station during the whole growing season. During the experiment, volumetric soil moisture was continually monitored every 10 min using soil moisture (ThetaProbe ML2x, Delta‐T Devices) sensors installed at a depth of 15 cm. Precipitation was measured by a rain gauge Rain‐O‐Matic Meteorological (Pronamic). During the experiment, volumetric soil moisture had been reduced at the end of the drought period relatively by 56, 61, and 42% in WD plots in 2012–2014, respectively, compared with the control treatment (C; 100%; Figure 1). The lower reduction in soil moisture in WD treatments in 2012 and 2013 was due to a drier summer during these years (Table 1, Figure S1).

For the characterization of hydrological conditions, we used the Emberger aridity index (AI) (Emberger, 1932). AI depends on the precipitation P (mm), the mean temperature *T*_c_ (°C) of the coldest month, and the mean temperature *T*_h_ (°C) of the hottest month within a specific time period (from April till the end of IDP in the present study). The index is defined as follows: AI = 1000 × P/(*T*_h_^2^ – *T*_c_^2^). In addition, we calculated water balance (WB), which is defined as the difference between precipitation P (mm) and potential evapotranspiration PET (mm). PET was estimated using the Penman–Monteith method, and it was calculated using 30 min data sets of air temperature, humidity, wind speed, solar (incident global radiation), and net (the balance between incoming and outgoing energy at the top of the canopy) radiations (Allen et al., 1998).

### 2.4 Production parameters and biomass stoichiometry

The aboveground biomass (AB) was determined annually by harvesting all aboveground biomass at the end of the IDP when the biomass reached a seasonal maximum of the growing seasons (July 10, 2012, July 3, 2013, and July 3, 2014). The area 0.3 × 0.3 m was harvested per plot. AB was dried to a constant mass (at 60°C) and weighed. Simultaneously with AB sampling, belowground plant biomass (BB) was determined in all treatments with the coring method. Soil cores (9.4 cm in diameter, 15 cm depth, *n* = 3) were collected within each treatment at the end of the IDP of the respective year. Collected samples were washed in nylon bags and on sieves of 0.5 mm mesh size and dried to a constant weight. R/S ratios were assessed based on total below‐ and aboveground biomass (BB/AB).

An automatic analyzer Flash 2000 (Thermo Scientific) was used to determine the contents of C and N in aboveground biomass. The dried AB was carefully homogenized, and subsequently, ∼2 mg of AB samples was used for elemental analyses to determine relative contents of C and N per unit dry weight (%). In addition, total N content in AB per one m^2^ of soil surface, defined as N uptake, was calculated.

### 2.5 Spectral reflectance and thermal measurements

Measurement of spectral reflectance (350–2500 nm) at the canopy level was carried out at the end of the IDP (Table 1) using a FieldSpec 4 Hi‐Res spectroradiometer (ASD Inc.). The reflectance measurements were conducted from a distance of ca 0.8 m perpendicular to the canopy surface using a pistol grip twice for each plot and then averaged. Before each new plot, the reference spectrum was measured using the white Spectralon reflectance standard (Labsphere). The reflected radiances were directly converted to spectral reflectance within the RS3 Spectral Acquisition Software (ASD Inc). Subsequently, vegetation and chlorophyll indices were computed from spectral reflectance curves (the calculated indices are listed in Table S1).

Thermal imaging measurements were acquired around noon from a distance of ca 1 m using an infrared thermal camera SC 660 (Flir Systems). Approximately, 100 points from each image were selected manually to avoid the effect of pixels from the soil background. The canopy temperature difference (*T*_diff_; °C) was calculated as the difference between canopy (*T*_c_) and air (*T*_a_) temperatures (*T*_diff_ = *T*_c_ – *T*_a_).

### 2.6 Statistical analyses

Before calculating the analysis of variance, the data for individual parameters were tested for normality using the Kolmogorov–Smirnov test. Three‐way ANOVA analysis was used to test the effect of WD, UV radiation and year on AB, BB, total biomass, and nitrogen and carbon in AB. The Fisher's LSD post hoc test was used to analyze differences between means. Significance levels are reported in the figures and tables as a significant with *p < .05, **p < .01, and ***p < .001. Pearson correlation coefficients (*r*) were calculated to evaluate the power of relationships between climatic and production parameters (Table S2) and between production parameters and thermal and spectral parameters (Table S3). All statistical tests were done using Statistica 12 software (StatSoft).

To identify the variables that explained a higher proportion of the total variance, which could provide insight into the relationships among climatic, production, nutrient, thermal, and spectral reflectance parameters, a principal component analysis (PCA) was performed using R 3.5.1 (R Core Team, 2014). In addition, to evaluate the interactive effect of WD and UV, linear regression was used to relate measured (i.e., observed) values (in the *y*‐axis) vs. calculated (i.e., predicted) values (in the *x*‐axis) for individual variables (Figure 6; Piñeiro et al., 2008). The predicted effect was the sum of percentage changes in response to WD and UV effects relative to the control. This predicted effect was compared with the measured combined WD + UV effect relative to the control. In these figures, the root mean square error (RMSE) was calculated for the 1:1 line.

## 3 RESULTS

### 3.1 Production characteristics

**FIGURE 2:**
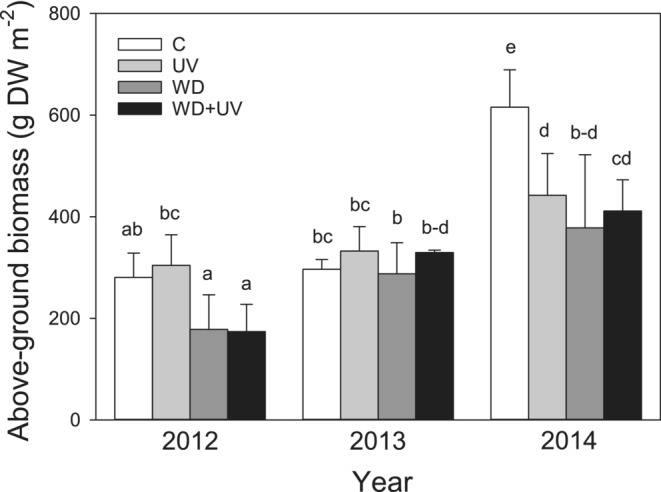
Effect of water deficit (WD), UV radiation (UV), and combined effect WD + UV on aboveground biomass in comparison with the control (C) in mountain grassland during years 2012–2014. Means (bars) and standard deviations (error bars) are presented (*n* = 3). Different letters denote statistically significant differences between treatments using Fisher's LSD post hoc test (*p* ≤ .05).

The ANOVA analysis confirmed the significant effect of WD on all production parameters and the significant effect of UV on BB, R/S, and TB (Table 2). A significant reduction in AB was observed in all treatments (WD, UV, and WD + UV) when compared to C in 2014 (Figure 2). Similar reductions were found in 2012 under WD and WD + UV treatments, but these declines were statistically not significant. In addition, a significant effect of year on AB was found (Table 2). AB was significantly higher in the year 2014 than in previous years, particularly in 2012 but not for WD treatments (Figure 2).

**TABLE 2:** Effects of water deficit (WD), UV radiation (UV), year (Y), and their interactions on production parameters (AB, aboveground biomass; BB, belowground biomass; R/S, root/shoot ratio; TB, total biomass) in mountain grassland in 2012–2014.

Effects	*df*	AB	BB	R/S	TB
WD	1	13.3***	10.5**	15.2***	5.3*
UV	1	0.2^ns^	5.5*	4.8*	6.0*
Y	2	34.2***	0.1^ns^	6.8**	2.4^ns^
WD × UV	1	2.0^ns^	1.1^ns^	1.7^ns^	0.4^ns^
Y × WD	2	3.3^ns^	1.7^ns^	1.8^ns^	1.1^ns^
Y × UV	2	1.9^ns^	2.2^ns^	0.5^ns^	3.3^ns^
Y × WD × UV	2	2.5^ns^	3.0^ns^	1.9^ns^	1.9^ns^

*Note*: *F*‐values of three‐way analysis of variance (ANOVA); ns, not significant, *p ≤ .05, **p ≤ .01, ***p ≤ .001.

**FIGURE 3:**
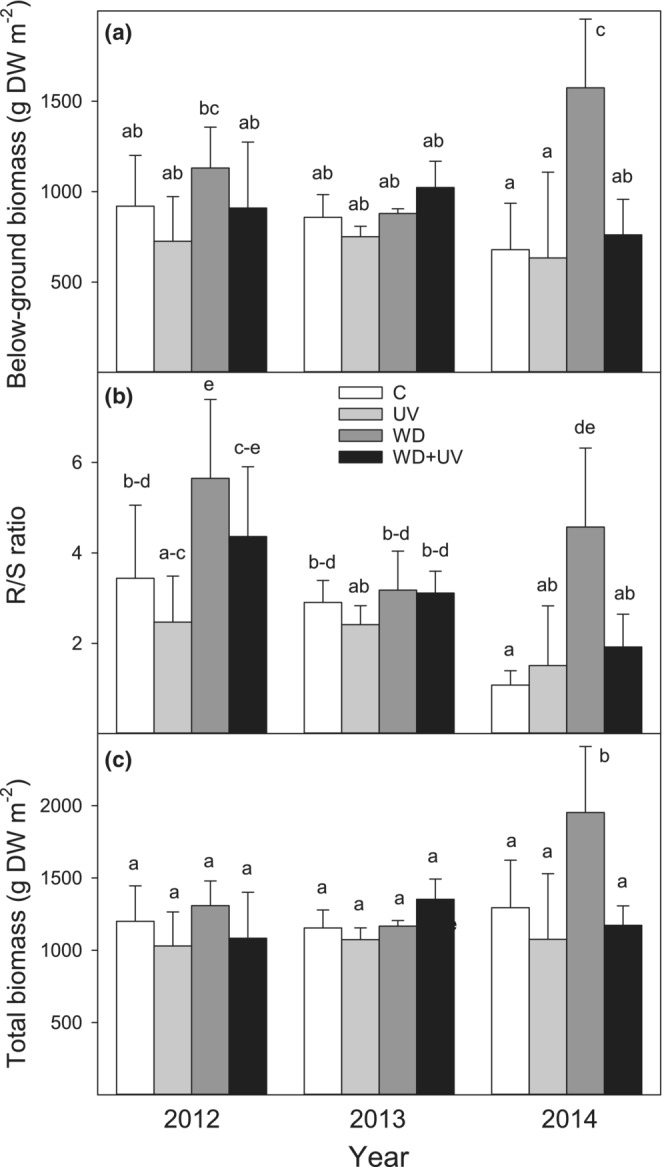
Effect of water deficit (WD), UV radiation (UV), and combined effect WD + UV on belowground biomass (a), R/S ratio (b), and total biomass (c) in comparison with control (C) in mountain grassland during years 2012–2014. Means (bars) and standard deviations (error bars) are presented (*n* = 3). Different letters denote statistically significant differences between treatments using Fisher's LSD post hoc test (*p* ≤ .05).

Generally, WD treatment resulted in a higher accumulation of BB in comparison with C and UV treatments, which led to a higher R/S ratio and TB (Figure 3a–c). Except in 2013, the R/S ratio was significantly higher under WD treatment (4.6–5.6) when compared to C (1.1–3.4). Also, the combined WD + UV treatment resulted in higher R/S values than C treatment, but these differences were statistically not significant (Figure 3b). Generally, differences in TB were nonsignificant during the whole experiment with an exception of a significant WD‐induced increase in 2014 (Figure 3c). None of the biomass parameters was affected by the interaction of WD, UV, and the year (Table 2).

**FIGURE 4:**
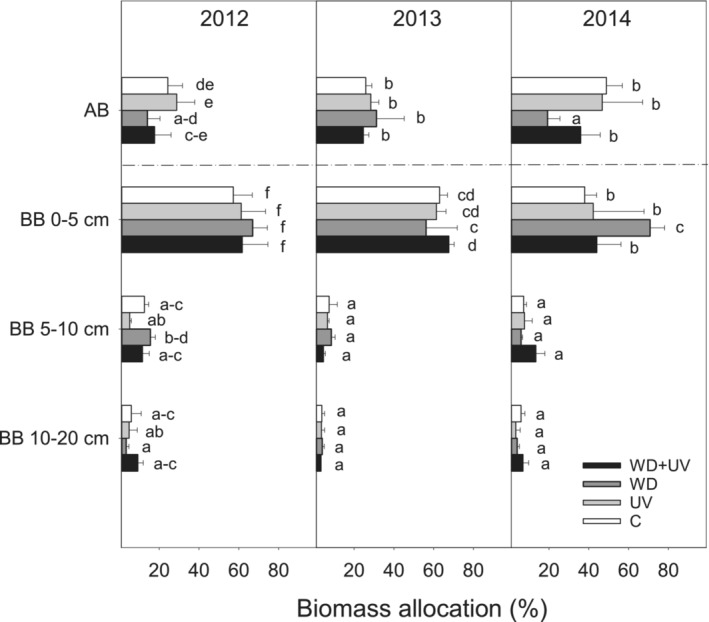
Effect of water deficit (WD), UV radiation (UV), and combined effect WD + UV on relative allocation of biomass in comparison with control (C) in mountain grassland in 2012–2014. Means (bars) and standard deviations (error bars) are presented (*n* = 3). Different letters denote statistically significant differences between treatments using Fisher's LSD post hoc test (*p* ≤ .05). AB, aboveground biomass; BB, belowground biomass.

A major part of TB was allocated to BB within soil horizon 0–5 cm, while the smallest fraction of biomass was allocated to soil horizon 5–10 cm and deeper (Figure 4). After 3 years of WD treatment (2014), biomass allocation to BB within soil horizon 0–5 cm significantly increased at the expense of AB. However, such response was not observed in the WD + UV treatment (Figure 4). In treatments without WD (C and UV), a higher biomass allocation to AB was found in 2014 (47–49%) in comparison with the previous 2 years, 2012 and 2013 (24–29%).

### 3.2 Nitrogen accumulation and C/N stoichiometry

**FIGURE 5:**
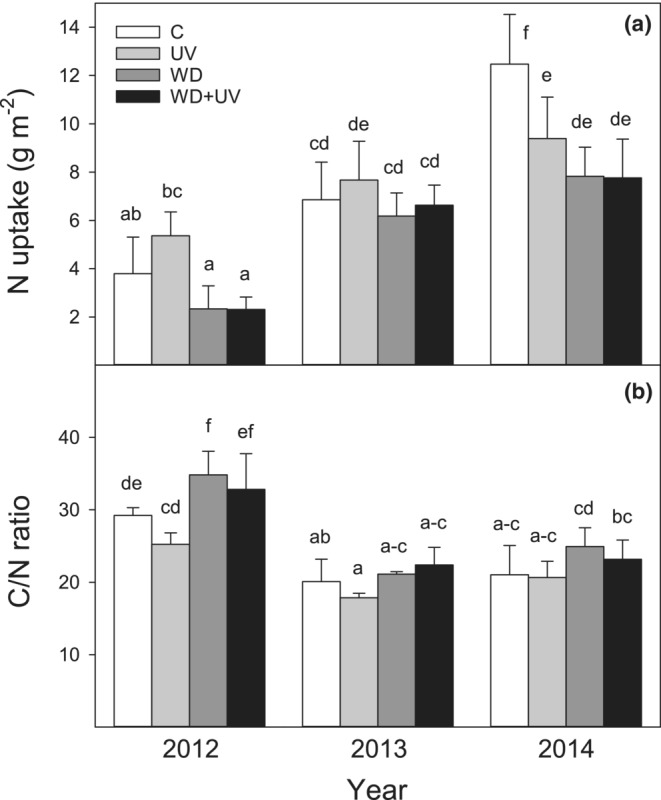
Effect of water deficit (WD), UV radiation (UV), and combined effect of WD + UV on nitrogen uptake per area (a) and C/N ratio (b) in aboveground biomass of mountain grassland in comparison with control (C) during years 2012–2014. Means (bars) and standard deviations (error bars) are presented (*n* = 3). Different letters denote statistically significant differences between treatments using Fisher's LSD post hoc test (*p* ≤ .05).

The ANOVA analysis confirmed the significant effect of WD on N content in AB, N uptake, and C/N ratio (Table 3). Generally, N content in AB was significantly lower under WD and WD + UV treatments (1.4–2.0%) when compared to C and UV treatments (1.8–2.6%) (Figure S2). Also, N uptake in AB was reduced in response to WD and WD + UV treatments when compared to C; however, it was significant only in 2014 (Figure 5a). The C/N ratio increased under WD compared with C conditions, but it was statistically significant again only in 2012 (Figure 5b).

**TABLE 3:** Effects of water deficit (WD), UV radiation (UV), year (Y), and their interactions on aboveground N content (N), aboveground C content (C), N uptake (total N content in aboveground biomass per unit area of soil; g m^−2^), and C/N ratio in mountain grassland in 2012–2014.

Effects	*df*	N (%)	C (%)	N uptake (g m^−2^)	C/N
WD	1	14.5***	0.0^ns^	21.0***	21.2***
UV	1	1.3^ns^	0.0^ns^	0.0^ns^	2.8^ns^
Y	2	34.0***	14.3***	56.9***	46.3***
WD × UV	1	1.5^ns^	1.9^ns^	0.2^ns^	0.6^ns^
Y × WD	2	0.0^ns^	0.1^ns^	2.1^ns^	1.7^ns^
Y × UV	2	0.3^ns^	1.9^ns^	2.8^ns^	0.7^ns^
Y × WD × UV	2	1.2^ns^	0.0^ns^	2.3^ns^	0.6^ns^

*Note*: *F*‐values of three‐way analysis of variance (ANOVA); ns, not significant, *p ≤ .05, **p ≤ .01, ***p ≤ .001.

No significant effect of UV radiation on nitrogen accumulation and C/N stoichiometry was observed (Table 3). Nevertheless, N uptake in AB was significantly reduced by UV treatment in 2014 when compared to C treatment (Figure 5a). No effect of UV radiation on the C/N ratio was observed throughout the study (Figure 5b).

### 3.3 Interactive effects of water deficit and UV radiation

**FIGURE 6:**
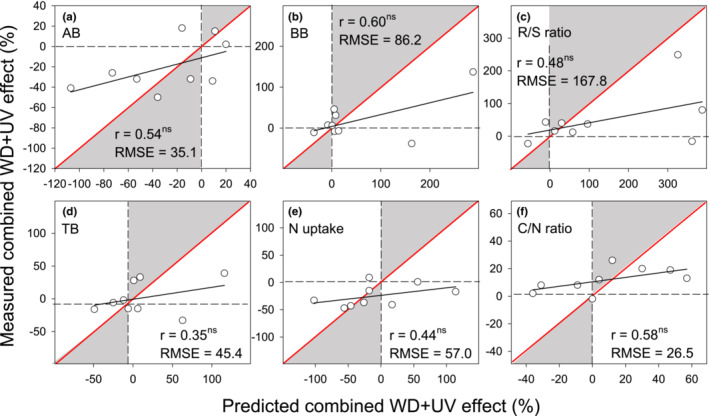
Relationship between observed (measured effect) and calculated (predicted effect) combined effect of WD and UV radiation for production parameters (AB, aboveground biomass; BB, belowground biomass; TB, total biomass, root: shoot, R/S ratio) and nutrient characteristics measured in AB. The predicted effect was calculated as the sum of individual WD and UV effects. The data were fitted using linear regression (best linear fit). Coefficients of determination (*r*) and significance levels (ns, *p* > .05) are shown. Root mean square error (RMSE) was calculated for the 1:1 line (red). Gray areas indicate more than additive or synergistic effects, while white areas indicate less than additive or antagonistic effects.

To evaluate the combined effect of WD and UV, we compared the predicted effect (the sum of the relative changes in individual WD and UV treatments compared with C treatment) with the measured effect of WD + UV treatment (compared with C treatment) for selected production and nutrient parameters. The relationship between measured and predicted effects of WD + UV treatment had lower slopes for all parameters when compared to theoretical 1:1 line indicating a less than additive (i.e., mutually alleviating) effect of both factors. This means that the real effect of combined factors is lower in both directions, negative and positive, than the sum of both factors acting separately (Figure 6).

### 3.4 Associations among environmental, production, and remote sensing parameters

**FIGURE 7:**
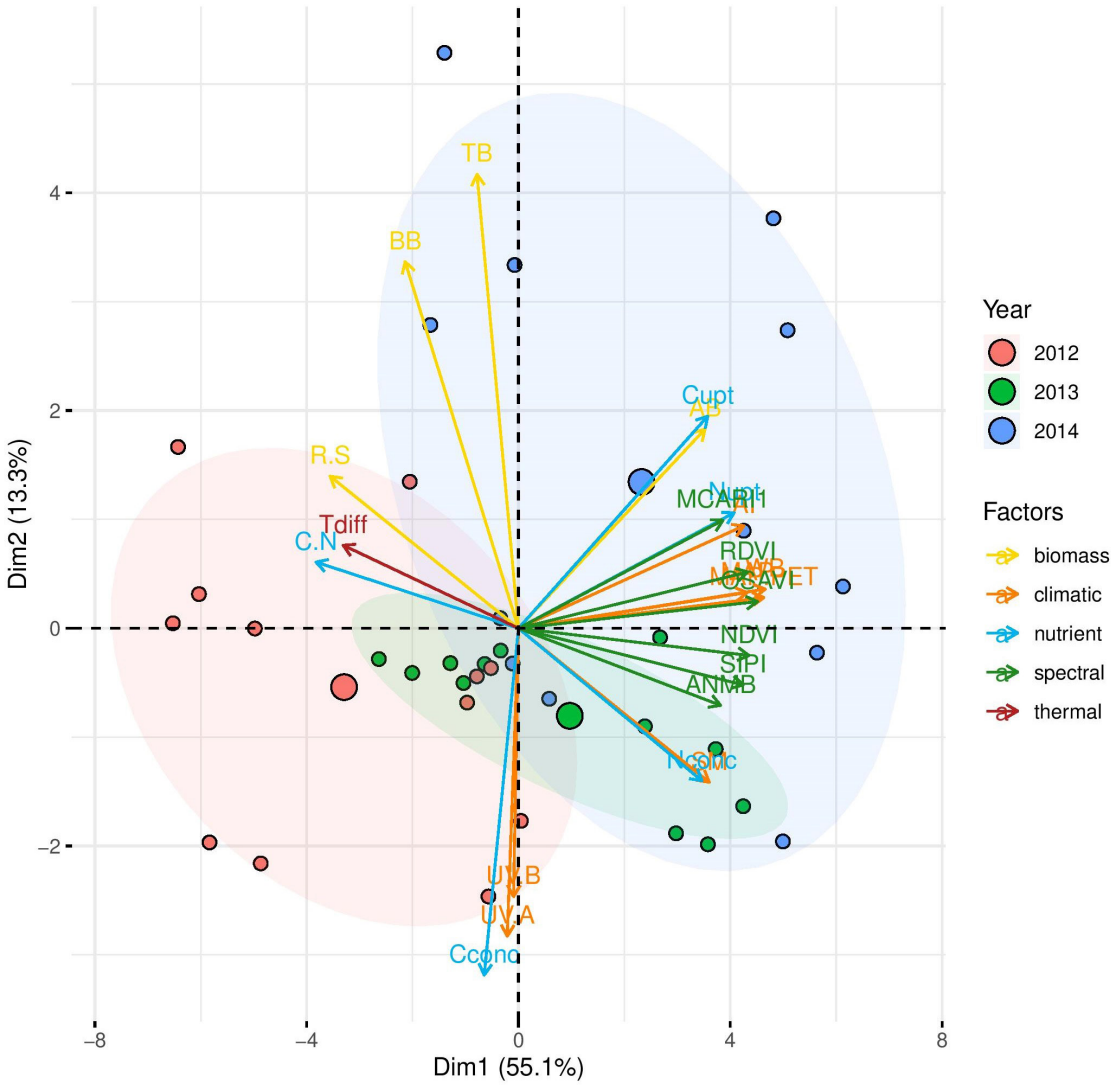
Principal component analysis (PCA) results for effects of precipitation (Prec), soil moisture (SM), aridity index (AI), water balance (WB), and UV radiation (UV‐A, UV‐B) on biomass parameters (AB, aboveground biomass; BB, belowground biomass; TB, total biomass; R.S, R/S ratio), nutrient parameters (Nconc, nitrogen (N) concentration in AB; Cconc, carbon (C) concentration in AB; Nupt, N uptake in AB per area; Cupt, C uptake in AB per area; C.N, C/N ratio in AB), spectral reflectance parameters (ANMB, ANMB_650‐725_, NDVI, RDVI, MCARI1, OSAVI, and SIPI), and thermal characteristics (Tdiff, *T*_diff_) in mountain grassland during 2012–2014.

To find potential associations that would explain the observed effects of WD and UV, and also potential remote sensing indicators for their estimation, a principal component analysis (PCA) based on all measured climatic, production, elemental, thermal infrared, and spectral reflectance parameters was performed (Figure 7). PCA revealed that AB, N content, and N uptake in AB were positively related to precipitation and other corresponding climatic parameters (soil moisture, aridity index, and water balance). In contrast, strong antagonistic relationships between R/S ratio and C/N ratio in AB and climatic parameters were observed (Figure 7).

PCA also revealed a strong positive association between UV radiation and C content in AB and negative association between UV radiation and TB. The best predictors for estimating changes in R/S and C/N ratios are *T*_diff_ (direct proportion) and NDVI, SIPI, and ANMB_650‐725_ (indirect proportion). The AB is best estimated using RDVI and MCARI1 spectral indices (Figure 7). In addition, a significant positive relationship between C/N and R/S ratios was observed (Figure S3).

**FIGURE 8:**
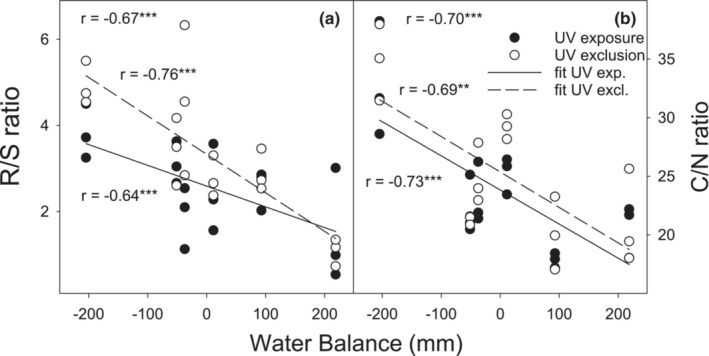
Relationships between the R/S ratio (a) or C/N ratio (b) and water balance in the mountain grassland during 2012–2014. The linear functions were fitted separately for both UV radiation treatments. Points represent individual replicates. Coefficients of determination (*r*) and significance levels (***p ≤ .001; **p ≤ .01) are shown.

AB significantly increased with increasing water regime parameters except for soil moisture. In addition, the significant negative relationships between BB and R/S ratio and water regime parameters were observed (Table S2). Moreover, the highest correlation coefficient (*r* = −0.67, *p* ≤ .001) for R/S ratio was found in the relationship with water balance (Figure 8a). Strong negative correlations between R/S ratio and all water regime parameters were also found separately in both UV exposure and UV exclusion treatments except soil moisture, where the significant relationship with R/S ratio was observed only in UV exclusion treatment. Moreover, N concentration and N uptake were significantly positively correlated with water regime parameters, while a negative relationship between C/N ratio and water regime parameters was found. The most significant negative correlation coefficient for C/N ratio was observed with water balance in both UV radiation treatments (Figure 8b).

### 3.5 Thermal imaging and spectral reflectance measurements

**FIGURE 9:**
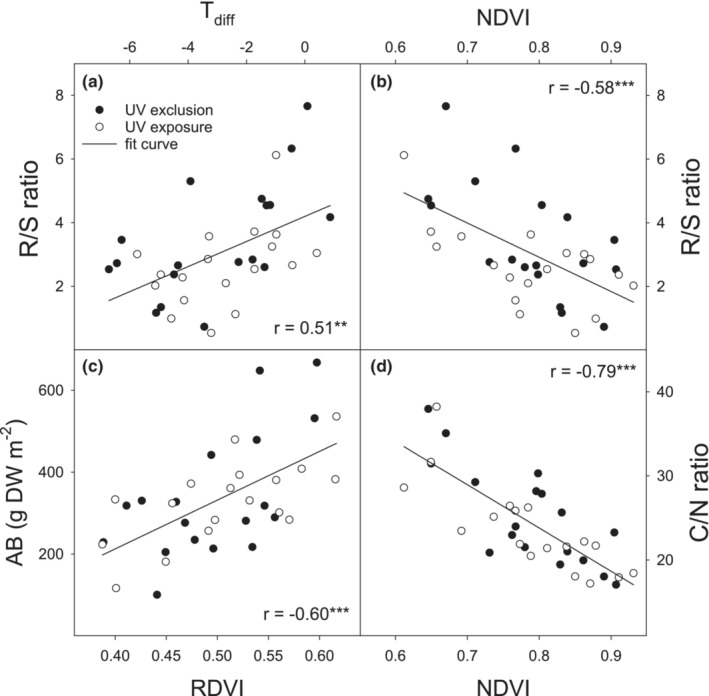
Relationships between selected production (aboveground biomass—AB and R/S ratio) or nutrient (C/N ratio) parameters and infrared thermography (*T*_diff_) and selected vegetation indices (NDVI and RDVI) in the mountain grassland during 2012–2014. The linear function was fitted together for both UV radiation treatments. Points represent individual replicates. Coefficients of determination (*r*) and significance levels (***p ≤ .001; **p ≤ .01) are shown.

Results of thermal infrared imaging showed that *T*_diff_ was significantly influenced by WD, but not by UV radiation (Table S3). Nevertheless, we found a significant increase in canopy temperature (reduction in canopy temperature difference) in response to UV compared with C treatment in 2013 (Figure S4). The main effect on *T*_diff_, however, had WD treatments, when *T*_diff_ increased by more than 80% and 69% (averaged over 2012–2014) under WD and WD + UV treatments, respectively, when compared to C treatment. The correlation analysis of thermal infrared imaging data showed significant relationships between *T*_diff_ and AB, BB, and R/S ratio as well as N uptake and C/N ratio (Table S4). The highest correlation coefficient was observed between *T*_diff_ and R/S ratio (Figure 9a).

All vegetation indices, except TCARI, TCARI/OSAVI, and NPQI, showed statistically significant responses to WD treatment, while these responses to UV radiation were negligible (Table S3). In addition, the correlation analysis which has been conducted for spectral reflectance indices and production parameters with data for each plot individually showed a significant correlation of 19 and 20 vegetation indices from a total of 24 calculated with AB and R/S ratio, respectively (Table S4). The highest correlation coefficients were observed between AB and RDVI and between R/S ratio and NDVI (Figure 9b,c). We have found only three significant correlations between BB and spectral reflectance indices (NDGI, VOG2, and GM1) and even no significant correlation with TB (Table S4).

Spectral reflectance indices were also tightly correlated with C and particularly N contents. Twenty‐two out of 24 vegetation indices tested significantly correlated with N content in AB, and 21 indices correlated with N uptake and C/N ratio (Table S4). On the contrary, only four significant correlations were found between C content in AB and vegetation indices. The most significant relationship between C/N ratio and NDVI is shown in Figure 9d.

## 4 DISCUSSION

### 4.1 Production characteristics

Extreme drought events affect numerous soil, plant, and ecosystem properties of grasslands and ultimately influence their productivity, biological diversity (Fay et al., 2008; Knapp et al., 2002; Kreyling et al., 2008), and also their ability to take up and utilize the nutrients (Grange et al., 2021; Kubert et al., 2021). In the present study, we found a significant negative effect of WD on the productivity of mountain grassland. In contrast, no marked effects of reduced precipitation by 50% on the aboveground productivity of grasses and forbs were found in the same mountain site during the water availability manipulation experiment in 2006–2008 (Holub et al., 2013). They found that the cover of dominant species such as *Nardus stricta*, *Festuca rubra*, *Avenella flexuosa*, *Potentilla erecta*, and *Vaccinium myrtillus* did not change in response to reduced precipitation (Holub et al., 2013). It is evident from their work that a mere 50% reduction in precipitation did not change the AB of the mountain grassland, which has, on average, a high ambient precipitation. It is in accordance with findings from a manipulative experiment with six precipitation treatments (Zhang et al., 2017). They observed that only the most extreme drought treatment (1/12 of annual precipitation) reduced the aboveground productivity in an alpine meadow and it was caused mainly by the reduction in forbs. On the contrary, Johnson et al. (2011) reported that repeated drought events reduced grass biomass, increased forb biomass, and led to an overall decrease in AB compared with controls in alpine snowbed community with dominant *Nardus stricta*.

On the contrary, the effect of UV radiation on the AB was not statistically significant in our study, which is consistent with findings of other authors, who summarized that the effects of UV‐B on plant biomass production are mostly species‐specific (e.g., Deckmyn & Impens, 1999). However, it is evident that UV radiation further modulates the effect of WD. While AB was in our experiment reduced by 36, 3, and 39% in response to WD, it was reduced by 43, 1, and 7% in response to combined WD + UV effect in 2012, 2013, and 2014, respectively. This marked difference in the last year of the experiment pointed to the positive effect of UV radiation on AB production under reduced water availability. Drought tolerance can be enhanced by the effect of UV radiation due to various reasons. Some authors suggest an interaction between drought stress and UV radiation through flavonoid biosynthesis (Nogués et al., 1998). Flavonoids play an important role as antioxidants and can mitigate the oxidative stress induced by drought stress. Rapantová et al. (2016) observed higher accumulation of flavonoids under the combined effect of WD and UV in grass as well as forbs.

Some authors mentioned that WD led to a significant increase in the fine root length density (root length in soil volume) and dry weight (e.g., Rodrigues et al., 1995; Walter et al., 2011). Robson et al. (2015), in a review of the literature, observed that UV radiation can alleviate the WD effect by higher root development and an increase in R/S ratio. In the present study, we found higher accumulation of BB in response to WD; however, we did not observe any significant change in R/S ratio in response to UV. Rapantová et al. (2016) observed species‐specific differences in response to UV radiation in the same drought experiment. They found that UV radiation alleviated the negative impact of WD in *A. capillaris*, *H. mollis*, and *H. maculatum*, while the additive effect of UV was observed in *R. obtusifolius* in response to WD. These different species‐specific effects can probably explain the lack of a significant positive effect of UV on biomass allocation to belowground plant parts in response to WD at the level of whole plant community in the present study. Thus, the first hypothesis that UV radiation alleviates the negative impact of WD through enhanced belowground biomass was not supported in our study.

### 4.2 N and C stoichiometry

In the present paper, N cycling was significantly affected by WD. The annually recurrent WD periods led to increasing C/N ratio in AB and decreasing N content and N uptake in AB in comparison with control. Gleeson et al. (2010) indicated that nitrification, in terms of both process rates and microbial populations responsible for nitrification activity, is highly responsive to soil water availability. Thus, water stress resulted in an increase of ammonium in the soil, which can hardly be taken up by plants. Alternating drying and wetting cycles lead to controversial effects. Although the alternated drying is often reported to increase N mineralization (Lu et al., 2020; Xiang et al., 2008), the cumulative N mineralization is mostly smaller under alternated drying and wetting than optimum soil moisture (Borken & Matzner, 2009). The effect of drying cycles on N mineralization also strongly depends on soil texture: in fine‐textured soils, the effect of drying is less pronounced due to nonlinear response (Austin et al., 2004). Jentsch et al. (2011) suggested that the composition of different microbial groups in soils remained unchanged in response to drought except for arbuscular mycorrhizal fungi. They proposed that an increase in C/N ratio in plants is caused by a lower microbial activity and reduced soil respiration under drought conditions, which can result in decreased rate of decomposition (Jentsch et al., 2011).

The effect of elevated UV‐B on N metabolism is often caused by changes in nitrite or nitrate reductase activity and reduction in leaf C/N ratio under elevated UV‐B suggesting competition between sucrose synthesis and nitrate reduction (Singh et al., 2015). However, no significant effect of UV radiation on nutrient characteristics was found in the present study.

### 4.3 Thermal imaging and spectral reflectance measurements

Thermal imaging represents one of the well‐established methods for indirect and noninvasive estimation of stomatal conductance and responses to WD (Jones, 1999). However, the final effect of WD on aboveground biomass can be different from stomatal response. Decline in stomatal conductance can be in the short‐term a sign of higher water use efficiency and thus lower negative response to drought stress; however, in the long term, it means a severe drop in biomass productivity (Blum, 2005). In the present study, thermal infrared imaging proved that canopy temperature increases due to WD. The UV effect was, however, lower than the WD effect and statistically insignificant. Under ambient precipitation, higher UV radiation led to an increase in the canopy temperature in comparison with control treatment. These results indicate that UV generally stimulated stomatal closure, but this stimulation was higher under sufficient water availability. *T*_diff_ also indicated the changes in C/N and R/S, which are mostly caused by WD.

Here, almost all selected vegetation indices showed statistically significant responses to WD. This is probably because the reflectance is strongly influenced by reduction in biomass and therefore changes in pigment composition are accentuated by differences in biomass. Correlation analysis for spectral reflectance indices and production parameters (AB and R/S ratio) showed the highest correlation for AB with RDVI and R/S ratio with NDVI or NDGI. Various authors reported different vegetation indices as most suitable for detection responses to drought stress. These include indices related to biomass or leaf area (NDVI, Aparicio et al., 2000), xanthophyll cycle carotenoids (PRI, Elsheery & Cao, 2008), water content (WI, Peñuelas et al., 1997), or accumulation of oxidized phenolic compounds (BPI, Peñuelas et al., 2004). The many indices shown to be sensitive to drought stress are probably caused by the occurrence of different stages and severity of drought stress within individual studies. Significant changes in aboveground biomass under severe drought stress can then mask the effect on pigment composition. This is likely the case also of our study where very similar responses were observed for different vegetation indices.

## 5 CONCLUSIONS

Water deficit (WD) during the main vegetation period (May–July) changes biomass allocation between above and belowground parts of our temperate mountain grassland. While aboveground biomass was reduced, belowground biomass increased, which led to higher R/S ratio in response to WD. However, the hypothesis that UV radiation alleviates the negative effect of WD through enhanced belowground biomass was not supported. Noticeably, WD led to reduced N uptake and increased C/N, but the interaction with UV radiation was also negligible. On the contrary, the results provide strong support for the hypothesis that vegetation indices based on thermal imaging and spectral reflectance (particularly NDVI and RDVI) can detect a reduction of aboveground biomass induced by drought conditions and consequently estimate changes in R/S ratio.

## AUTHOR CONTRIBUTIONS

**Petr Holub:** Conceptualization (equal); data curation (equal); investigation (equal); validation (equal); visualization (equal); writing – original draft (equal). **Karel Klem:** Conceptualization (equal); methodology (equal); validation (equal); writing – original draft (equal). **Barbora Veselá:** Data curation (supporting); investigation (equal); writing – original draft (supporting). **Kateřina Surá:** Data curation (supporting); investigation (equal); validation (supporting). **Otmar Urban:** Funding acquisition (lead); methodology (supporting); supervision (equal); writing – original draft (equal).

## CONFLICT OF INTEREST

The authors declare that they have no conflict of interest.

## Supporting information


Figure S1

Figure S2

Figure S3

Figure S4

Table S1–S4

## Data Availability

The data that support the findings of this study are openly available in Mendeley: “Holub_etal_EE_dataset,” Mendeley Data, V1, https://doi.org/10.17632/d79khnmhkf.1. The data are available at https://data.mendeley.com/datasets/d79khnmhkf/draft?a=86d777df‐5837‐488a‐a616‐8c57fcd66260.
