# Supplementary figures and images for: Interactive effects of UV radiation and water deficit on production characteristics in upland grassland and their estimation by proximity sensing
Source: Ecol Evol. 2022 Sep 23;12(9):e9330. doi: 10.1002/ece3.9330 (PMC9502068; doi:10.1002/ece3.9330)

Precipitation (mm)

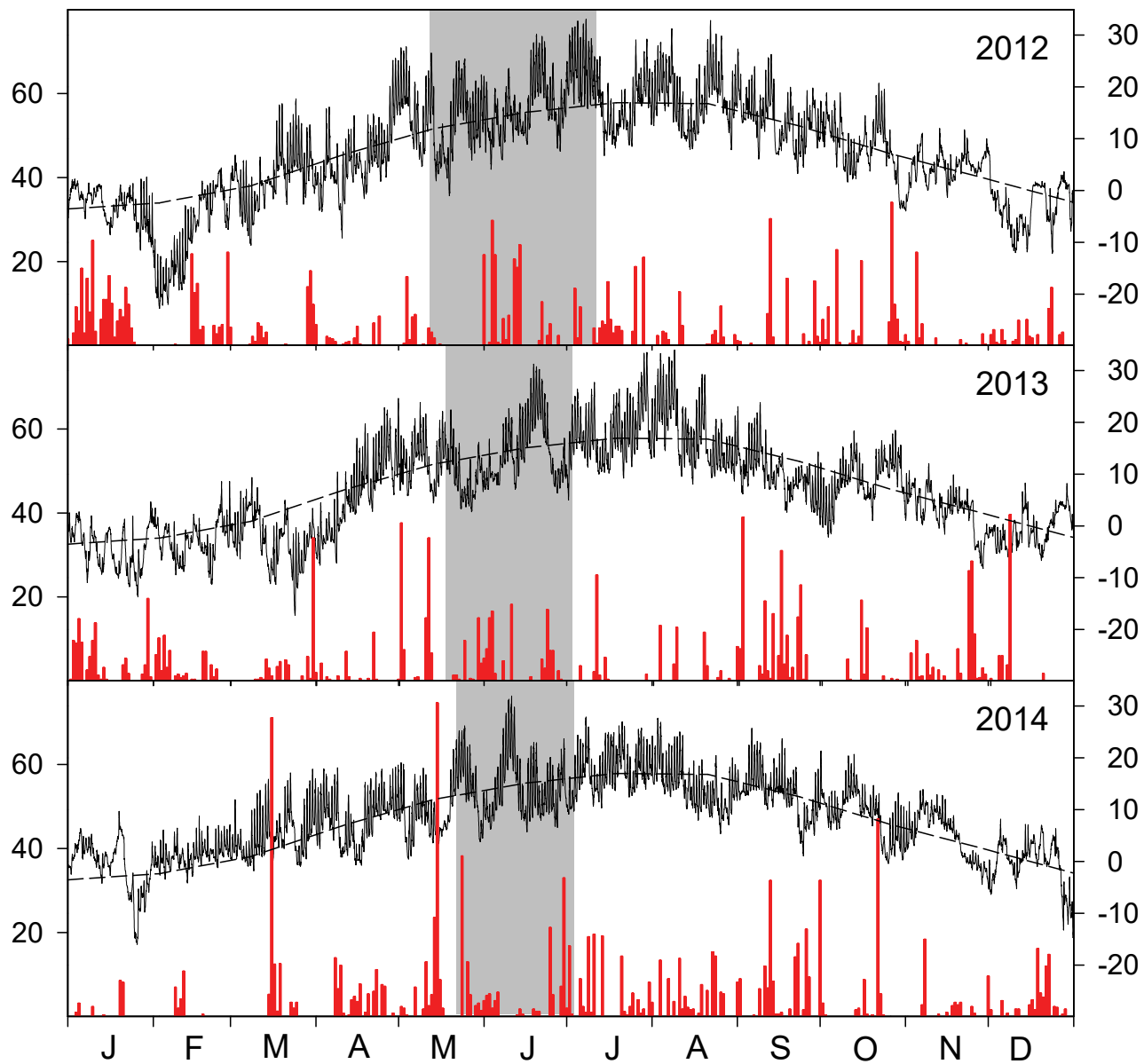

Month

Air temperature (°C)

Supplement: Supplementary file 1 — Figure S1 [file ECE3-12-e9330-s005.pdf]

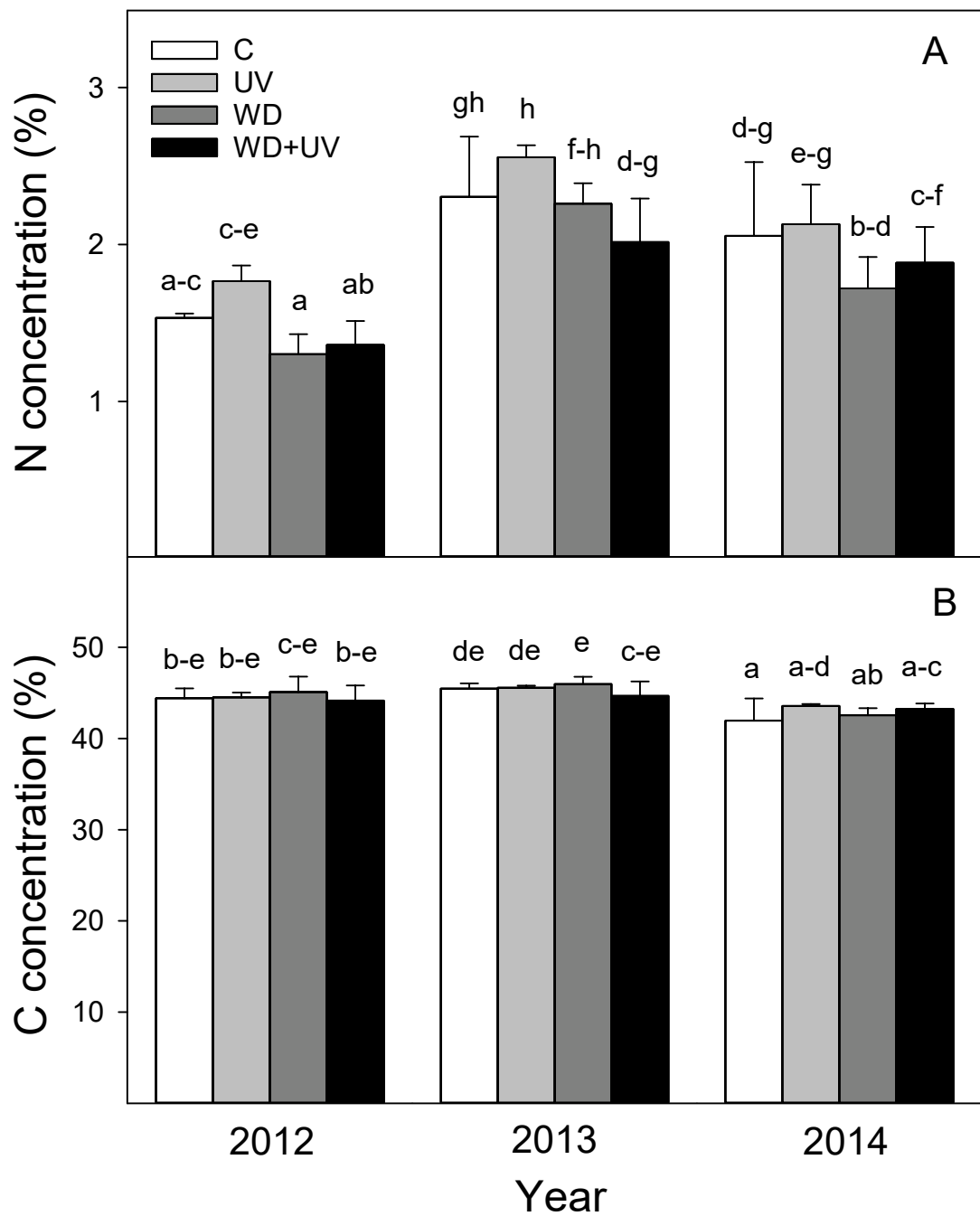

Supplement: Supplementary file 2 — Figure S2 [file ECE3-12-e9330-s004.pdf]

C/N ratio

$r = 0.54^{***}$

40

30

20

0

2

4

6

8

R/S ratio

- UV exclusion
- UV exposure
- fit curve

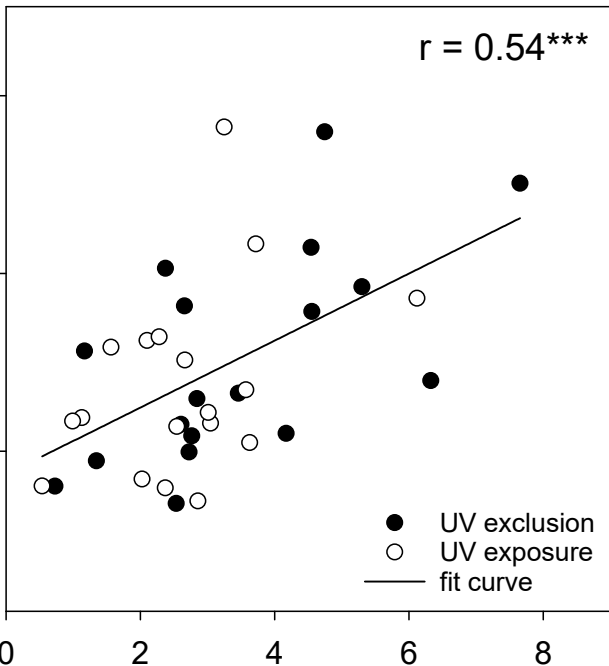

Supplement: Supplementary file 3 — Figure S3 [file ECE3-12-e9330-s003.pdf]

Canopy temperature difference (°C)

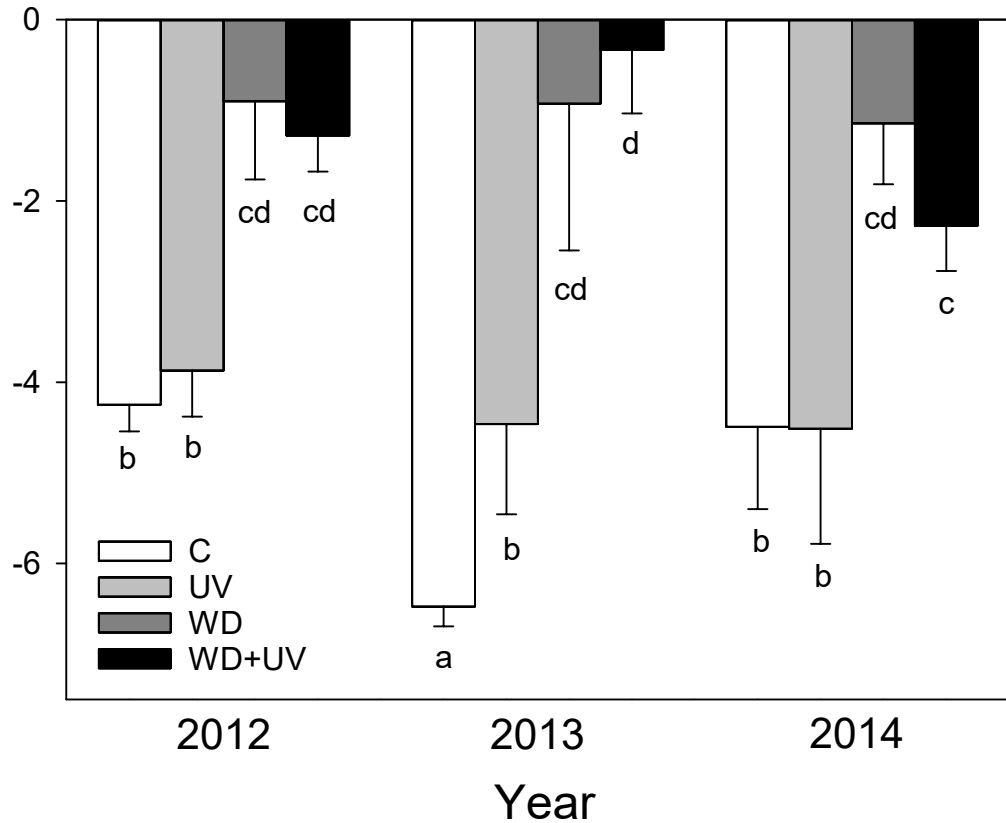

Supplement: Supplementary file 4 — Figure S4 [file ECE3-12-e9330-s001.pdf]
