# Supplementary material for: Interactive effects of UV radiation and water deficit on production characteristics in upland grassland and their estimation by proximity sensing
Source: Ecol Evol. 2022 Sep 23;12(9):e9330. doi: 10.1002/ece3.9330 (PMC9502068; doi:10.1002/ece3.9330)
Supplement: Supplementary file 5 — Table S1–S4 [file ECE3-12-e9330-s002.docx]

**Appendix**

(see Tables S1–S4 and Figs. S1–S4)

**Table S1.** Vegetation indices used, with equations for the calculations and references. R_xxx_ refers to the reflectance at wavelength xxx in nanometers.

**Table S2.** Pearson correlation coefficients (r) and their significances (****p* ≤ 0.001; ***p* ≤ 0.01; **p* ≤ 0.05; ns – not significant) for relationships between climatic and production parameters in mountain grassland in 2012–2014 (n = 36).

|  | AB | | BB | | R/S | | | TB | | |
| --- | --- | --- | --- | --- | --- | --- | --- | --- | --- | --- |
|  | r | p | r | p | r | p | r | | p |  |
| Precipitation | 0.68 | *** | -0.42 | * | -0.64 | *** | -0.16 | | n.s. |  |
| Soil moisture | 0.25 | n.s. | -0.38 | * | -0.45 | ** | -0.29 | | n.s. |  |
| Aridity index | 0.76 | *** | -0.37 | * | -0.64 | *** | -0.07 | | n.s. |  |
| Water balance | 0.71 | *** | -0.39 | * | -0.67 | *** | -0.12 | | n.s. |  |
| UV-A | -0.11 | n.s. | -0.32 | n.s. | -0.22 | n.s. | -0.37 | | * |  |
| UV-B | -0.03 | n.s. | -0.34 | * | -0.25 | n.s. | -0.36 | | * |  |
|  | N conc | | N uptake | | C conc | | | C/N | | |
|  | r | p | r | p | r | p | r | | p |  |
| Precipitation | 0.44 | ** | 0.69 | *** | -0.29 | n.s. | -0.54 | | *** |  |
| Soil moisture | 0.61 | *** | 0.36 | * | 0.19 | n.s. | -0.59 | | *** |  |
| Aridity index | 0.40 | * | 0.77 | *** | -0.41 | * | -0.52 | | ** |  |
| Water balance | 0.60 | *** | 0.78 | *** | -0.24 | n.s. | -0.70 | | *** |  |
| UV-A | 0.01 | n.s. | -0.10 | n.s. | 0.02 | n.s. | -0.04 | | n.s. |  |
| UV-B | -0.06 | n.s. | -0.06 | n.s. | -0.08 | n.s. | 0.01 | | n.s. |  |

**Table S3.** Effects of water deficit (WD), UV radiation (UV), year (Y) and their interactions on infrared thermography (*T*_diff_) and spectral reflectance parameters in mountain grassland in 2012–2014. F-values of three-way analysis of variance (ANOVA); ns - not significant, **p* ≤ 0.05, ***p* ≤ 0.01, ****p* ≤ 0.001.

| Effects | WD | UV | Y | WD x UV | Y x WD | Y x UV | Y x WD x UV |
| --- | --- | --- | --- | --- | --- | --- | --- |
| *df* | 1 | 1 | 2 | 1 | 2 | 2 | 2 |
| *T*_diff_ | 158.4^***^ | 0.8 ^n.s.^ | 1.4 ^n.s.^ | 3.8 ^n.s.^ | 5.4^*^ | 3.9^*^ | 0.1 ^n.s.^ |
| ANMB_650-725_ | 57.2^***^ | 0.2 ^n.s.^ | 44.2^***^ | 0.4 ^n.s.^ | 3.4^*^ | 2.0 ^n.s.^ | 0.8 ^n.s.^ |
| NDVI | 71.5^***^ | 0.0 ^n.s.^ | 58.1^***^ | 0.0 ^n.s.^ | 1.1 ^n.s.^ | 0.9 ^n.s.^ | 0.1 ^n.s.^ |
| NDGI | 40.1^***^ | 0.8 ^n.s.^ | 19.3^***^ | 0.0 ^n.s.^ | 0.7 ^n.s.^ | 0.4 ^n.s.^ | 0.0 ^n.s.^ |
| NRERI | 9.7^**^ | 0.6 ^n.s.^ | 5.0^*^ | 0.1 ^n.s.^ | 0.7 ^n.s.^ | 0.7 ^n.s.^ | 0.1 ^n.s.^ |
| RDVI | 42.5^***^ | 0.9 ^n.s.^ | 30.5^***^ | 1.7 ^n.s.^ | 0.9 ^n.s.^ | 0.1 ^n.s.^ | 0.1 ^n.s.^ |
| MSR | 57.3^***^ | 0.2 ^n.s.^ | 35.9^***^ | 0.1 ^n.s.^ | 4.5^*^ | 0.7 ^n.s.^ | 0.3 ^n.s.^ |
| MCARI1 | 21.3^***^ | 0.5 ^n.s.^ | 22.0^***^ | 1.9 ^n.s.^ | 1.0 ^n.s.^ | 0.1 ^n.s.^ | 0.1 ^n.s.^ |
| TCARI | 0.3 ^n.s.^ | 0.0 ^n.s.^ | 7.0^*^ | 0.6 ^n.s.^ | 0.1 ^n.s.^ | 0.2 ^n.s.^ | 0.1 ^n.s.^ |
| OSAVI | 61.4^***^ | 0.5 ^n.s.^ | 45.7^***^ | 1.0 ^n.s.^ | 0.8 ^n.s.^ | 0.2 ^n.s.^ | 0.1 ^n.s.^ |
| TCARI/OSAVI | 1.8 ^n.s.^ | 0.1 ^n.s.^ | 4.8^*^ | 0.5 ^n.s.^ | 0.1 ^n.s.^ | 0.3 ^n.s.^ | 0.1 ^n.s.^ |
| Grenness Index | 48.1^***^ | 0.2 ^n.s.^ | 42.8^***^ | 0.2 ^n.s.^ | 7.4 ^n.s.^ | 0.6 ^n.s.^ | 0.8 ^n.s.^ |
| TVI | 21.0^***^ | 0.6 ^n.s.^ | 19.7^***^ | 2.1 ^n.s.^ | 1.0 ^n.s.^ | 0.1 ^n.s.^ | 0.1 ^n.s.^ |
| ZM | 40.5^***^ | 0.2 ^n.s.^ | 24.5^***^ | 0.0 ^n.s.^ | 2.3 ^n.s.^ | 1.0 ^n.s.^ | 0.3 ^n.s.^ |
| SRPI | 32.4^***^ | 0.1 ^n.s.^ | 36.4^***^ | 0.1 ^n.s.^ | 3.6^*^ | 1.5 ^n.s.^ | 0.5 ^n.s.^ |
| NPQI | 0.4 ^n.s.^ | 0.9 ^n.s.^ | 5.2^*^ | 0.3 ^n.s.^ | 0.5 ^n.s.^ | 0.4 ^n.s.^ | 0.2 ^n.s.^ |
| PRI | 33.8^***^ | 0.4 ^n.s.^ | 19.8^***^ | 0.0 ^n.s.^ | 0.8 ^n.s.^ | 1.8 ^n.s.^ | 0.6 ^n.s.^ |
| NPCI | 32.8^***^ | 0.0 ^n.s.^ | 43.4^***^ | 0.0 ^n.s.^ | 3.1 ^n.s.^ | 1.7 ^n.s.^ | 0.4 ^n.s.^ |
| SIPI | 65.7^***^ | 0.0 ^n.s.^ | 45.2^***^ | 0.0 ^n.s.^ | 1.4 ^n.s.^ | 0.6 ^n.s.^ | 0.1 ^n.s.^ |
| VOG3 | 25.1^***^ | 0.6 ^n.s.^ | 13.5^***^ | 0.0 ^n.s.^ | 1.1 ^n.s.^ | 0.8 ^n.s.^ | 0.1 ^n.s.^ |
| VOG2 | 24.8^***^ | 0.6 ^n.s.^ | 13.4^***^ | 0.0 ^n.s.^ | 0.9 ^n.s.^ | 0.8 ^n.s.^ | 0.1 ^n.s.^ |
| GM1 | 16.5^***^ | 2.3 ^n.s.^ | 3.2 ^n.s.^ | 0.0 ^n.s.^ | 0.6 ^n.s.^ | 0.2 ^n.s.^ | 0.1 ^n.s.^ |
| GM2 | 48.0^***^ | 0.1 ^n.s.^ | 29.4^***^ | 0.1 ^n.s.^ | 3.9^*^ | 1.0 ^n.s.^ | 0.5 ^n.s.^ |
| WI | 20.8^***^ | 0.5 ^n.s.^ | 23.9^***^ | 0.0 ^n.s.^ | 2.3 ^n.s.^ | 0.2 ^n.s.^ | 0.1 ^n.s.^ |
| WI/NDVI | 43.6^***^ | 0.3 ^n.s.^ | 36.5^***^ | 0.0 ^n.s.^ | 5.1^*^ | 0.8 ^n.s.^ | 0.0 ^n.s.^ |

**Table S4.** Pearson correlation coefficients (r) and their significances (****p* ≤ 0.001; ***p* ≤ 0.01; **p* ≤ 0.05; ns – not significant) for relationships between infrared thermography (*T*_diff_) or spectral reflectance indices and production or nutrient parameters in mountain grassland in 2012–2014 (n = 36).

|  | AB | | BB | | R/S | | TB | |
| --- | --- | --- | --- | --- | --- | --- | --- | --- |
|  | r | p | r | p | r | p | r | p |
| T_diff_ | -0.37 | * | 0.37 | * | 0.51 | ** | 0.23 | n.s. |
| ANMB_650-725_ | 0.35 | * | -0.24 | n.s. | -0.44 | ** | -0.11 | n.s. |
| NDVI | 0.52 | ** | -0.30 | n.s. | -0.58 | *** | -0.10 | n.s. |
| NDGI | 0.45 | ** | -0.35 | * | -0.58 | *** | -0.18 | n.s. |
| NRERI | 0.31 | n.s. | -0.29 | n.s. | -0.43 | ** | -0.17 | n.s. |
| RDVI | 0.60 | *** | -0.28 | n.s. | -0.52 | ** | -0.04 | n.s. |
| MSR | 0.39 | * | -0.30 | n.s. | -0.49 | ** | -0.15 | n.s. |
| MCARI1 | 0.59 | *** | -0.22 | n.s. | -0.44 | ** | 0.02 | n.s. |
| TCARI | 0.38 | * | 0.02 | n.s. | -0.12 | n.s. | 0.18 | n.s. |
| OSAVI | 0.59 | *** | -0.29 | n.s. | -0.56 | *** | -0.06 | n.s. |
| TCARI/OSAVI | 0.06 | n.s. | 0.20 | n.s. | 0.20 | n.s. | 0.23 | n.s. |
| Greennes Index | 0.35 | * | -0.23 | n.s. | -0.41 | * | -0.09 | n.s. |
| TVI | 0.58 | *** | -0.22 | n.s. | -0.43 | ** | 0.01 | n.s. |
| ZM | 0.40 | * | -0.30 | n.s. | -0.49 | ** | -0.14 | n.s. |
| SRPI | 0.51 | ** | -0.26 | n.s. | -0.51 | ** | -0.06 | n.s. |
| NPQI | 0.19 | n.s. | -0.11 | n.s. | -0.22 | n.s. | -0.03 | n.s. |
| PRI | 0.41 | * | -0.32 | n.s. | -0.50 | ** | -0.16 | n.s. |
| NPCI | -0.53 | *** | 0.26 | n.s. | 0.52 | ** | 0.05 | n.s. |
| SIPI | 0.47 | ** | -0.31 | n.s. | -0.56 | *** | -0.13 | n.s. |
| VOG3 | -0.41 | * | 0.33 | n.s. | 0.51 | ** | 0.17 | n.s. |
| VOG2 | -0.41 | * | 0.33 | * | 0.52 | ** | 0.17 | n.s. |
| GM1 | 0.28 | n.s. | -0.36 | * | -0.48 | ** | -0.26 | n.s. |
| GM2 | 0.36 | * | -0.28 | n.s. | -0.46 | ** | -0.14 | n.s. |
| WI | 0.58 | *** | -0.31 | n.s. | -0.54 | *** | -0.08 | n.s. |
| WI/NDVI | -0.46 | ** | 0.25 | n.s. | 0.54 | *** | 0.08 | n.s. |
|  | N conc | | N uptake | | C conc | | C/N | |
|  | r | P | r | p | r | p | r | p |
| T_diff_ | -0.32 | n.s. | -0.36 | * | -0.02 | n.s. | 0.36 | * |
| ANMB_650-725_ | 0.77 | *** | 0.56 | *** | 0.25 | n.s. | -0.72 | *** |
| NDVI | 0.77 | *** | 0.70 | *** | 0.06 | n.s. | -0.79 | *** |
| NDGI | 0.73 | *** | 0.64 | *** | 0.15 | n.s. | -0.73 | *** |
| NRERI | 0.59 | *** | 0.49 | ** | 0.28 | n.s. | -0.54 | *** |
| RDVI | 0.61 | *** | 0.71 | *** | -0.25 | n.s. | -0.69 | *** |
| MSR | 0.75 | *** | 0.60 | *** | 0.16 | n.s. | -0.72 | *** |
| MCARI1 | 0.48 | ** | 0.65 | *** | -0.37 | * | -0.58 | *** |
| TCARI | 0.04 | n.s. | 0.30 | n.s. | -0.56 | *** | -0.19 | n.s. |
| OSAVI | 0.71 | *** | 0.74 | *** | -0.13 | n.s. | -0.77 | *** |
| TCARI/OSAVI | -0.33 | n.s. | -0.08 | n.s. | -0.53 | *** | 0.21 | n.s. |
| Greennes Index | 0.73 | *** | 0.55 | *** | 0.11 | n.s. | -0.69 | *** |
| TVI | 0.46 | ** | 0.63 | *** | -0.38 | * | -0.57 | *** |
| ZM | 0.74 | *** | 0.61 | *** | 0.20 | n.s. | -0.71 | *** |
| SRPI | 0.70 | *** | 0.69 | *** | 0.01 | n.s. | -0.69 | *** |
| NPQI | 0.36 | * | 0.33 | n.s. | 0.24 | n.s. | -0.31 | n.s. |
| PRI | 0.72 | *** | 0.61 | *** | 0.13 | n.s. | -0.70 | *** |
| NPCI | -0.71 | *** | -0.71 | *** | -0.01 | n.s. | 0.71 | *** |
| SIPI | 0.77 | *** | 0.66 | *** | 0.12 | n.s. | -0.77 | *** |
| VOG3 | -0.70 | *** | -0.61 | *** | -0.20 | n.s. | 0.67 | *** |
| VOG2 | -0.70 | *** | -0.61 | *** | -0.20 | n.s. | 0.67 | *** |
| GM1 | 0.56 | *** | 0.45 | ** | 0.25 | n.s. | -0.53 | *** |
| GM2 | 0.74 | *** | 0.57 | *** | 0.21 | n.s. | -0.70 | *** |
| WI | 0.66 | *** | 0.74 | *** | -0.11 | n.s. | -0.70 | *** |
| WI/NDVI | -0.71 | *** | -0.61 | *** | -0.11 | n.s. | 0.73 | *** |

**Figure S1.** Daily precipitation (bars) and 30 min air temperature (full line) in comparison with long term average 1998–2014 (dashed line) over three experimental years 2012–2014. Gray background indicates the induced drought period.

**Figure S2.** The effect of water deficit (WD), UV radiation (UV), and combined effect of WD+UV on nitrogen (A) and carbon (B) concentrations in above-ground biomass of mountain grassland in comparison with control (C) during years 2012–2014. Means (bars) and standard deviations (error bars) are presented (n = 3). Different letters denote statistically significant differences between treatments using Fisher LSD post-hoc test (p ≤ 0.05).

**Figure S3**. The relationships between the R/S ratio and C/N ratio in above-ground biomass in the mountain grassland during 2012–2014. Points represent individual replicates. Coefficient of determination (r) and significance level (***p ≤ 0.001) is shown.

**Figure S4.** The effect of water deficit (WD), UV radiation (UV), and combined effect of WD+UV on canopy temperature difference (difference between canopy and air temperatures) in comparison with control (C) in mountain grassland during years 2012–2014. Means (bars) and standard deviations (error bars) are presented (n = 3). Different letters denote statistically significant differences between treatments using Fisher LSD post-hoc test (p ≤ 0.05).
